# Contrasting Torpor Use by Reproductive Male Common Noctule Bats in the Laboratory and in the Field

**DOI:** 10.1093/icb/icad040

**Published:** 2023-05-26

**Authors:** Lara Keicher, J Ryan Shipley, Paul J Schaeffer, Dina K N Dechmann

**Affiliations:** Max Planck Institute of Animal Behavior, Am Obstberg 1, 78315 Radolfzell, Germany; Department of Biology, University of Konstanz, Universitätsstraße 10, 78457 Konstanz, Germany; Swiss Federal Institute for Forest, Snow, and Landscape Research WSL, Zürcherstraße 111, Birmensdorf 8903 CH, Switzerland; Department of Biology, Miami University, 700 E. High St., Oxford, OH 45056, USA; Max Planck Institute of Animal Behavior, Am Obstberg 1, 78315 Radolfzell, Germany; Department of Biology, University of Konstanz, Universitätsstraße 10, 78457 Konstanz, Germany; Centre for the Advanced Study of Collective Behaviour, Universitätsstraße 10, 78457 Konstanz, Germany

## Abstract

Metabolic processes of animals are often studied in controlled laboratory settings. However, these laboratory settings often do not reflect the animals’ natural environment. Thus, results of metabolic measurements from laboratory studies must be cautiously applied to free-ranging animals. Recent technological advances in animal tracking allow detailed eco-physiological studies that reveal when, where, and how physiological measurements from the field differ from those from the laboratory. We investigated the torpor behavior of male common noctule bats (*Nyctalus noctula*) across different life history stages using two approaches: in controlled laboratory experiments and in the field using calibrated heart rate telemetry. We predicted that non-reproductive males would extensively use torpor to conserve energy, whereas reproductive males would reduce torpor use to promote spermatogenesis. We did not expect differences in torpor use between captive and wild animals as we simulated natural temperature conditions in the laboratory. We found that during the non-reproductive phase, both captive and free-ranging bats used torpor extensively. During reproduction, bats in captivity unexpectedly also used torpor throughout the day, while only free-ranging bats showed the expected reduction in torpor use. Thus, depending on life history stage, torpor behavior in the laboratory was markedly different from the wild. By implementing both approaches and at different life history stages, we were able to better explore the limitations of eco-physiological laboratory studies and make recommendations for when they are an appropriate proxy for natural behavior.

## Introduction

Animal behavior is shaped by a myriad of physiological, social, and environmental factors. To better understand the relationship between behavioral and physiological responses and the factors that shape them, both laboratory and field studies are needed. Controlled laboratory studies provide the accurate measurement of physiological processes that often guide the ideas behind general principles; however, the controlled conditions are only a proxy for what animals experience in the wild (Geiser et al. 2000; Costa and Sinervo 2004). In contrast, field studies observe the free-ranging animals in their complex natural environment, limiting the ability to link single parameters to variation in behavior (Costa and Sinervo 2004). Consequently, the interpretation of the results of field studies is often more challenging. Findings from laboratory and field experiments will frequently differ, and additional investigation is needed to understand the mechanisms underlying a behavior (Geiser et al. 2000). In this sense, laboratory and field studies are complementary, and provide the tools and data to disentangle the mechanisms that shape different behavioral and physiological strategies (Calisi and Bentley 2009).

Torpor is one of the most effective energy-saving mechanisms in heterothermic mammals, marsupials, and birds (Geiser 2021), and its use can differ significantly between laboratory studies and natural environments (Geiser et al. 2000). Torpor is defined as a controlled reduction of metabolic rate, during which heart rate is decreased and body temperature conforms to or near to ambient temperature (Geiser 2004). In addition to considerable energetic savings, torpor has various other functions, such as water conservation, fat storage prior to migration, or removal of parasites (Nowack et al. 2017). Under laboratory conditions, flow-through respirometry at a range of environmental temperatures is the most accurate method to quantify torpor and its relationship to energy use (Lighton 2018). In free-ranging animals, torpor is often monitored using proxies for internal body temperature, either via skin temperature (Chruszcz and Barclay 2002; Willis et al. 2006; Webb and Mzilikazi 2010; Brigham et al. 2011; Kobbe et al. 2011; Körtner et al. 2016) or thermal imaging (Shankar et al. 2022). However, skin temperature-based methods can miss torpid states, especially during periods of thermoconformity at higher ambient temperatures (Reher and Dausmann 2021; Keicher et al. 2022). In contrast, heart rate is tightly coupled with the energy metabolism of animals, reflects immediate changes in energy expenditure (Currie et al. 2014), and can be used to follow patterns of torpor use in natural environments remotely (Dechmann et al. 2011; O'Mara et al. 2017a, 2017b). Recently, devices have become small enough to allow remote tracking of the heart rate of small animals. This has revealed torpor strategies where heart rate and skin temperatures can be decoupled in both tropical bat species (O'Mara et al. 2017a, 2017b) and temperate-zone bat species (Keicher et al. 2022).

Torpor use in free-ranging animals is thought to be highly flexible and modulated by many environmental factors, such as ambient temperature (Buck and Barnes 2000; Matheson et al. 2010), photoperiod (Adams 2010; Smit et al. 2011; Shipley et al. 2015), or time of the day (Geiser et al. 2007), as well as internal factors such as physiological or reproductive state (Grinevitch et al. 1995; Dietz and Kalko 2006; Dzal and Brigham 2013; Körtner et al. 2016), body condition (Webb and Mzilikazi 2010; Kobbe et al. 2011; Vuarin et al. 2013), feeding success (Stawski and Geiser 2011), or age (Rojas et al. 2014). Often, these factors are linked with each other, which makes the interpretation of field observations challenging. For example, food availability and foraging success are influenced by environmental conditions and ambient temperatures (Anthony et al. 1981; Waringer 1991; Peng et al. 1992; Burles et al. 2009; Hałat et al. 2018), all of which can directly translate to changes in torpor use. In contrast, most laboratory studies focus on torpor use by post-absorptive individuals from a single life history stage at manipulated ambient temperatures (Currie et al. 2018). This study design facilitates the comparison of results across studies, but rarely reflects the conditions animals would encounter in the wild. To understand torpor use and its evolutionary significance to fitness and survival, laboratory studies should simulate environmental conditions that are likely to promote realistic use of torpor as well as measure individuals at different life history stages (Keicher et al. 2022).

Insectivorous bats from the temperate zones use torpor regularly to conserve energy (Geiser 2021). Most research comparing torpor patterns between life history stages in free-ranging bats has been performed with females (Lausen and Barclay 2003; Willis et al. 2006; Dzal and Brigham 2013), as the cost of torpor, such as reduced fetal growth or milk production, has been long recognized (Audet and Fenton 1988; Hamilton and Barclay 1994; Grinevitch et al. 1995; Racey and Speakman 1987). However, it is becoming increasingly clear that in males, too, energetic costs increase during spermatogenesis (Racey 1974; Kurta and Kunz 1988; Hałat et al. 2020; Komar et al. 2020; Komar et al. 2022), during courtship, or when defending a harem (Entwistle et al. 1998; Speakman et al. 2003; Becker et al. 2013). This leads to reduced torpor use by males during these life history stages (Kurta and Kunz 1988; Gebhard 2013). As an alternative energy-conserving strategy, similar to reproductive females, males of some species may switch from a solitary lifestyle to roosting in temporary colonies in the summer (Safi 2008). Captive studies on male parti-colored bats (*Vespertilio murinus*) have shown that they are able to reduce torpor use and maintain high body temperatures more easily when kept in groups (E. Komar et al., submitted for publication), and free-ranging males during spermatogenesis are more likely to form colonies when insect abundance is lower and during colder weather (Hałat et al. 2018, 2020). Roosting in groups may then allow them to profit not only from more efficient foraging, but also from each other’s body temperature and/or from spending less energy on maintaining high body temperatures (Safi 2008) while avoiding torpor.

In this study, we investigated how a heterothermic species adjusts their energy management strategies across life history stages, comparing bats’ behavior in the laboratory and in the field. We worked with the common noctule bat (*Nyctalus noctula*), which is one of the few bat species from the temperate zone where males briefly form small colonies in summer during reproduction (Heise 1985; personal observation). *Nyctalus noctula* hibernates from November until March and has been observed to regularly use torpor throughout the annual life cycle. After hibernation, females from populations in southern Germany and Switzerland migrate in spring farther north or northeast to form maternity colonies (Dechmann et al. 2014; Lehnert et al. 2018; O'Mara et al. 2019), while the males do not migrate and stay at the hibernation site all year. Their reproductive season starts in May with the onset of spermatogenesis, and continues through the summer until mating in August/September as soon as the females return. Despite the regular use of torpor, pregnant females reduce torpor use when kept under semi-natural conditions in the laboratory (Keicher et al. 2022). We now predicted that males would also reduce or avoid torpor during reproduction to facilitate sperm production. We exposed male bats to a natural daily temperature cycle in the laboratory at four life history stages: (1) pre-reproduction (April), (2) early reproduction (June), (3) late reproduction (July), and (4) post reproduction (October) to understand patterns of torpor use. We hypothesized that “pre-reproduction” and “post reproduction” male bats would use torpor throughout the day and most of the night to save energy. In contrast, “early reproduction” and “late reproduction” male bats would avoid or minimize torpor use to decrease negative impacts on spermatogenesis. We then monitored the torpor use of free-ranging bats after release during “pre-reproduction” and “early reproduction” to investigate if the behavior in the laboratory matches the behavior in the field using calibrated heart rate telemetry.

## Methods

### Study animals

We worked with adult male common noctule bats (*N. noctula*) from a population in southern Germany (hereafter “Konstanz”, 47°39′59.8″N, 9°10′53.6″E). We removed the bats from bat boxes during the day or caught them emerging from a natural tree cavity at dusk with mist nets (Ecotone, Gdynia, Poland) and transported them in individual soft cloth bags to the nearby Max Planck Institute of Animal Behavior. We studied bats during four life history stages: non-reproductive male bats just after hibernation (3–6 April 2019 and 7–20 April 2020; “pre-reproduction”), reproductive bats in early summer (12 June to 1 July 2020; “early reproduction”), reproductive bats in mid-summer (8–26 July 2019; “late reproduction”), and non-reproductive bats before hibernation (2–15 October 2019; “post reproduction”; see Supplementary Table S1 for an overview). Dates for experiments were chosen based on previous data from regular population monitoring, in which reproductive stages had been recorded based on size of testes and *C. epididymes*. At different stages, size of testes and cauda epididymes changed (see below), as bats underwent spermatogenesis. Bats in stage “pre-reproduction” and “early reproduction” were released with the heart rate transmitters still attached (see below), and their torpor use was monitored in the field.

### Body mass, body condition, and food intake

After transport to the laboratory, we weighed bats with a digital scale (± 0.01 g; Kern and Sohn, Bahlingen, Germany). Bats received mealworms and water *ad libitum* during their natural foraging time at dusk, and the weight of consumed mealworms was measured. We weighed bats every day before and after each experiment (see below), and bats maintained their body mass while in captivity.

### Size of testes and cauda epididymes

In *N. noctula*, spermatogenesis and filling of testicles does not start until May and proceed during the summer (Racey 1974). Bats from April had flat testicles, indicating no ongoing spermatogenesis, and were defined as non-reproductive (“pre-reproduction”). To describe the progress of spermatogenesis, we used testicular and epididymal length, assessed through external measurement using digital calipers (± 0.01 mm, Mahr IP67 MarCal 16EW). Throughout June and July (“early” and “late reproduction”), the testicles were ball-shaped and increased in length, indicating ongoing spermatogenesis. Before mating in September and after the male colonies in the wild dissolve, sperm is transferred to the cauda epididymes. Note that our data thus included measurements of testes length (“pre-reproduction”, “early”, and “late reproduction”) and length of the cauda epididymes (“post reproduction”).

### Torpor-use experiments

We performed two experiments in the laboratory while simultaneously measuring oxygen consumption (V̇O_2_), heart rate (*f*_H_), and skin temperature (*T*_skin_) (see below).

“Laboratory-day” experiment: We placed the bats individually in respirometry chambers in a climate-controlled incubator sometime between 21:00 and 23:30 the night prior to the first experiment. Bats had then time to acclimate overnight until we began to measure V̇O_2_ and *f*_H_ in the following morning from 06:00 until 18:00. Bats did not have access to food while in the respirometry chambers. After the experiment, we fed bats with mealworms and placed them back into the respirometry chambers in the incubator, and continued to monitor *f*_H_ throughout the night (until 06:00). Incubator temperature was set based on ambient air temperatures (*T*_a_) from Konstanz from the previous two years (data from the Climate Data Center of the German Weather Service) during the corresponding month. We used calculated means for three 4-h time periods during the day (06:00–10:00, 10:00–14:00, and 14:00–18:00) and a single mean temperature during the night (18:00–06:00). Resulting incubator temperatures during “pre-reproduction” were 9.1, 13.6, 15.0, and 9.0°C, and during “early reproduction” 17.1, 20.9, 22.1, and 16.4°C. During “late reproduction”, incubator temperatures were 18.6, 22.6, 23.9, and 17.9°C, and during “post reproduction”, 9.1, 12.2, 12.8, and 9.2°C. The presence of the bats, especially when not torpid, affected the temperature in the small respirometry chambers. Thus, we measured laboratory temperatures (*T*_lab_) directly in the chambers and used these for analyses.

“Calibration” experiment: The following day we exposed the bats to a range of incubator temperatures in six increasing 1-h increments (0, 7.5, 15, 22.5, 27.5, and 32.5°C) from 06:00 to 12:00 (Supplementary Fig. S1). This was to calibrate V̇O_2_ and *f*_H_ of each individual at a larger range of *T*_lab_.

After a maximum of three days, bats were then returned to the box they had been removed from or released at the capture site in the evening of the “calibration” experiment. During “pre-reproduction” and “early reproduction” in 2020, we released bats with external heart rate transmitters (see below and supplemental material) and continuously measured their *f*_H_. The transmitter fell off after a maximum of seven days.

### Comparison of laboratory and field ambient temperatures

We obtained hourly *T*_a_ data from Konstanz from the Climate Data Center of the German Weather Service (DWD, https://opendata.dwd.de/climate_environment/CDC/) from the days we followed free-ranging bats in the field (“pre-reproduction” and “early reproduction”).

### Measurement of oxygen consumption, heart rate, and skin temperature

To measure metabolic rates, we used an open-flow pull-through respirometry system with humidity control (Sable Systems International, Las Vegas, NV, USA). We standardized individual rates of oxygen consumption with the mean of the body mass before and after experiments and report V̇O_2_ in mL O_2_ g^−1^ h^−1^. We continuously monitored *f*_H_ throughout the laboratory and field experiments using external heart rate transmitters (SP2000 HR, Sparrow Systems, Fisher, IL, USA). *T*_skin_ during the laboratory experiments was recorded every two minutes using modified iButtons. See supplementary material for a detailed description of procedures.

### Sociality and foraging behavior assessment

We assessed if bats roosted in a group or single by counting emerging bats during dusk. We monitored the number and duration of foraging flights per night of “pre-reproduction” and “early reproduction” bats using heart rate telemetry data. *f*_H_ from flying bats were easily distinguishable from roosting bats, as *f*_H_ increase to ∼900 bpm (Supplementary Fig. S2) and a general change in signal occurred. As soon as the bats returned to the roost, signal strength increased, and *f*_H_ signals were clearer, as the antenna on the transmitter moves less on a bat in the roost compared to in flight.

### Data analysis and statistics

We tested if daytime *T*_lab_ and nighttime *T*_lab_ were comparable with *T*_a_ conditions the bats experienced in the field using ANOVAs followed by pairwise comparisons using the Tukey HSD test (p-adjustment method “Holm”).

We compared mean body mass, mean testes length, and mean food intake after the “laboratory-day” experiment in male bats at the four life history stages using ANOVAs followed by pairwise comparisons using the Tukey HSD test (p-adjustment method “Holm”). Note that cauda epididymes, being a different organ, was not directly compared to testes.

We defined different physiological states (torpid, resting, arousal, and torpor entry) by either visually inspecting *f*_H_ and, if available, V̇O_2_ over time for each individual in the “laboratory-day” experiment (Keicher et al. 2022), during nights in captivity, and in the field (Supplementary Fig. S2). Experimental V̇O_2_ and *f*_H_ were highly correlated (*R*^2^ = 0.84) across all temperatures (“laboratory-day” and “calibration” experiments). We therefore used *f*_H_ to define different physiological states. In the field, we defined a “bat day” to start at sunrise and end when the bat emerged from the roost. A “bat night” started with emergence from the roost and ended at sunrise. We excluded the first day of recording after release to minimize the effect of captivity and handling on the bats’ behavior in the data.

We calculated the relative time spent in torpor (expressed as a percentage between 0 and 100) during the “laboratory-day” experiment, during the night recordings of *f*_H_ in captivity, and throughout the 24-h period in the field for each individual. In the field, we only included “bat days” and “bat nights” where at least two-thirds of the day or night had been recorded. After excluding the first day in the field and days/nights with insufficient *f*_H_ recordings, we analyzed eleven “bat days” from four “pre-reproduction” bats, and five “bat nights” from three “pre-reproduction” bats in the field. We analyzed ten “bat days” from four “early reproduction” bats and eleven “bat nights” from five “early reproduction” bats in the field. The average recording coverage was 91% for “bat days” and 84% for “bat nights”. We calculated the average relative time spent in torpor during “bat days” and “bat nights”, whenever we had recorded multiple days and nights for that individual.

We compared relative time spent in torpor (day and night) in the laboratory between the four life history stages using beta regression (betareg; Zeileis et al. 2010) followed by Tukey HSD tests for pairwise comparison (emmeans; Lenth 2021). We then compared the relative time spent in torpor between the laboratory and the field (day and night) in “pre-reproduction” and “early reproduction” bats using beta regression (glmmTMB; Magnusson et al. 2017) followed by Tukey HSD tests for pairwise comparison. We included BatID as a random effect to account for repeated measures. We compared mean flight duration per night during “pre-reproduction” and “early reproduction” using linear mixed models with batID as a random effect.

All analyses were performed in R (Version R 4.0.2; R Core Team 2022; RStudio Version 1.1.456; RStudio Team 2022).

## Results

### Comparison of laboratory and field ambient temperatures

Overall laboratory and field temperatures differed significantly (ANOVA *F*_3,49_ = 20.64, *P* < 0.001). In April 2020 (“pre-reproduction”), field *T*_a_ were exceptionally high (mean = 16.8 ± 2.9°C), and during the day field *T*_a_ were significantly higher than day *T*_lab_ (mean = 12.5 ± 0.8°C, *t* = −3.70, *P* < 0.01), while during the night field *T*_a_ (mean = 10.3 ± 2.5°C) and night *T*_lab_ (mean = 9.3 ± 0.4°C) did not differ (*t* = −1.30, *P* = 0.20). In contrast, in June, during “early reproduction” days *T*_lab_ (mean = 19.1 ± 1.0°C) and field *T*_a_ (mean = 20.6 ± 2.9°C) did not differ (*t* = −1.53, *P* = 0.13). “Early reproduction” night *T*_lab_ (mean = 17.9 ± 0.8°C) and night field *T*_a_ (mean = 16.3 ± 1.6°C) also did not differ (*t* = 2.27, *P* = 0.06). Overall, ambient temperatures during “early reproduction” were higher compared to “pre-reproduction”.

### Body mass, testes/cauda epididymes length, and food intake

Body mass differed significantly between the four life history stages and increased from “pre-” to “post reproduction” (ANOVA: *F*_3,31_ = 14.2, *P* < 0.001). Testes length increased significantly from “pre-reproduction” to “early” and then “late reproduction” (ANOVA: *F*_2,20_ = 81.21, *P* < 0.001) and presumably accounted to a large part of the body mass change. Pairwise comparisons (Tukey HSD test) indicated that during “early reproduction” testes were significantly larger than during “pre-reproduction” ( *t* = 4.58, *P* < 0.001), and then increased further until “late reproduction”, indicating ongoing spermatogenesis during “early” and “late reproduction”. The cauda epididymes, being a different organ, were not directly compared but were clearly filled when bats had reached “post reproduction”. Food intake after the “laboratory-day” experiment did not differ significantly across the four life history stages (ANOVA: *F*_3,31_ = 1.2, *P* = 0.3). See Table 1 for mean values and significant differences of body mass and food intake across the four life history stages.

**Table 1.** Sample size, mean body mass ± s.d., mean testes/cauda epididymes length ± s.d., and mean food intake ± s.d. across all four life history stages

	Sample size*	Body mass (g)	Testes/cauda epididymes length (mm)	Food intake (g)
Pre-reproduction	11 (7)	26.56 ± 1.56^a^	5.91 ± 0.98^a^	4.09 ± 2.40^a^
Early reproduction	9	27.36 ± 1.68^ab^	8.35 ± 1.06^b^	5.00 ± 1.39^a^
Late reproduction	7	29.24 ± 1.39^bc^	12.97 ± 1.12^c^	3.43 ± 1.32^a^
Post reproduction	8 (7)	31.05 ± 1.70^c^	9.55 ± 1.74**	4.32 ± 0.59^a^

* Sample sizes for testes/cauda epididymes measurements are in parentheses.

** In the “post reproduction” phase, the length of the cauda epididymes and not the testes was measured, and length of the cauda epididymes was not directly compared to testes length. Different superscript letters indicate significant differences (*P* < 0.01; Tukey HSD test).

### Torpor-use in the laboratory was lowest during “late reproduction”

**Fig. 1:**
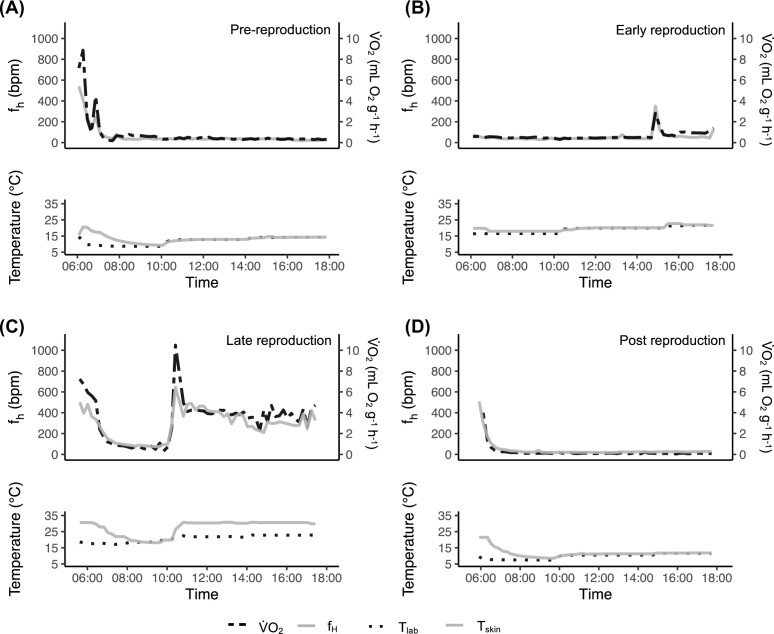
Exemplary plots of bats from the four life history stages in the “laboratory-day” experiment. In each panel, the upper plot shows *f*_H_ (gray solid line) and V̇O_2_ (black dashed line) over a 12-h time period. The lower plot shows *T*_skin_ (gray solid line) and *T*_lab_ (black solid line). (A) A “pre-reproduction” bat remains torpid with low *f*_H_ and V̇O_2_ after a short arousal at the beginning of the experiment. *T*_skin_ thermoconforms to *T*_lab_. (B) An “early reproduction” bat also remains torpid with low *f*_H_ and V̇O_2_, and *T*_skin_ thermoconforms to *T*_lab_. (C) A “late reproduction” bat enters torpor from 07:00 until 10:00. The rest of the day, the bat is non-torpid with an elevated *f*_H_ and V̇O_2_, and *T*_skin_ higher than *T*_lab_. (D) A “post reproduction” bat remains torpid throughout the day with low *f*_H_ and V̇O_2_, and *T*_skin_ thermoconforms to *T*_lab_. Overall, *f*_H_ and V̇O_2_ are decoupled from changes in *T*_skin_ and *T*_lab_ when bats are torpid.

**Fig. 2:**
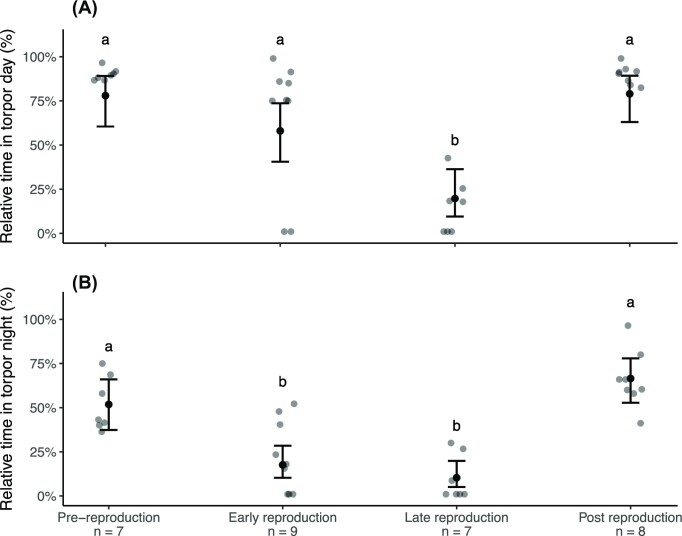
Torpor use during the “laboratory-day” experiment (A) and during the night (B) across the four life history stages. Predicted values with 95% confidence interval (bars) and raw data (dots) of relative time bats spent torpid across the four life history stages are presented. Different letters indicate significant differences (Tukey HSD test). Number of bats are given below each life history stage.

When bats used torpor in the laboratory, V̇O_2_ and *f*_H_ decreased to a minimum and remained low, despite a change in *T*_lab_ and consequently *T*_skin_ (Figs. 1A, B, and D, Supplementary Fig. S1). When not torpid, V̇O_2_ and *f*_H_ fluctuated, and *T*_skin_ was higher than *T*_lab_ (Fig. 1C). The relative time bats from all four life history stages spent torpid during the day differed significantly (Fig. 2A, Supplementary Table S2). “Late reproduction” bats used less daytime torpor than “pre-reproduction” (*z*-ratio = 5.56, *P* < 0.001), “early reproduction” (*z*-ratio = 3.40, *P* < 0.01), and “post reproduction” ( *z*-ratio = −5.90, *P* < 0.001) bats (Fig. 2A). During the night, too, the relative time bats spent torpid was significantly different across the four stages (Fig. 2B, Supplementary Table S2). After the evening feeding, bats usually did not enter torpor for at least 1 h, likely to digest the consumed food (Supplementary Fig. S2A and B). “Early reproduction” and “late reproduction” bats did not differ (*z*-ratio = 1.31, *P* = 0.56) in torpor use; they frequently aroused or rested and spent less time in torpor during the night compared to “pre-reproduction” and “post reproduction” bats that did not differ in torpor use ( *z*-ratio = −1.48, *P* = 0.45).

### “Early reproduction” bats used less torpor in the laboratory compared to the field

**Fig. 3:**
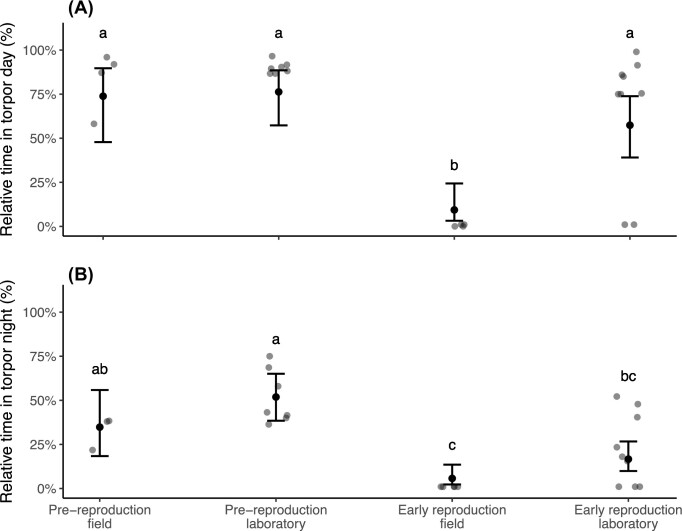
“Pre-reproduction” and “early reproduction” torpor use in the laboratory and field during the day (A) and night (B). Predicted values with 95% CI (bars) and raw data (dots) of relative time bats spent torpid in % during the laboratory experiments (laboratory) and field heart rate monitoring (field) during “pre-reproduction” (day: *n*_bats_ = 4, *n*_bat days_ = 11; night: *n*_bats_ = 3, *n*_bat nights_ = 5) and “early reproduction” (day: *n*_bats_ = 4, *n*_bat days_ = 10; night: *n*_bats_ = 5, *n*_bat nights_ = 11). Different letters indicate significant differences (Tukey HSD test).

During the day, free-ranging “early reproduction” bats spent less time in torpor compared to free-ranging “pre-reproduction” bats ( *t*-ratio = 3.88, *P* < 0.01) as well as captive “early reproduction” (*t*-ratio = −3.64, *P* < 0.01) and “pre-reproduction” bats (Fig. 3A, *t*-ratio = 4.43, *P* < 0.001). Specifically, three out of four “early reproduction” bats decreased the time spent torpid during the day from >75% in the laboratory to almost 0% in the field. The fourth individual never used torpor during the day, either in the laboratory or the field. Relative time spent in torpor during the day was the same in captive “early reproduction” bats and captive ( *t*-ratio = 1.51, *P* = 0.45) and free-ranging “pre-reproduction” bats (*t*-ratio = 1.09, *P* = 0.7). Similarly, the relative time spent torpid during the night did not differ in captive and free-ranging bats during “pre-reproduction” (*t*-ratio = −1.35, *P* = 0.55) and “early reproduction” (Fig. 3B, *t*-ratio = −2.22, *P* = 0.15). Overall, “early reproduction” bats spent less time torpid compared to “pre-reproduction” bats during the night in the laboratory and field.

### Sociality and foraging behavior

In the field, all “pre-reproduction” bats roosted alone, except one bat that roosted in a small group of up to ten individuals. “Pre-reproduction” bats performed one or two flights per night (mean number of flights per night = 1.25 ± 0.46), with a mean total flight duration per night of 57 ± 15 min. Only one “early reproduction” bat was always solitary; all other bats roosted in small groups between 25 and 30 individuals and performed one to four flights per night (mean number of flights per night = 2.28 ± 1.06) with a significantly longer mean total flight duration per night of 106 ± 32 min compared to “pre-reproduction” bats (*z* = 2.93, *P* = 0.003). “Late reproduction” and “post reproduction” bats were not observed in the field.

## Discussion

We investigated how reproductive stage influences energy management strategies in male *N. noctula* and found that sperm-producing bats used less daytime torpor. In addition, although bats were under simulated natural conditions in the laboratory, we found that the frequency of torpor use varied significantly between observations in captivity and the wild.

As predicted, non-reproductive captive male bats spent the largest amount of time torpid during the day. Torpor use decreased throughout reproduction alongside ongoing spermatogenesis (Fig. 2), and daytime torpor use reached a minimum during the period of maximal testes size in “late reproduction”. During this stage, spermatozoa had not yet been transferred to the cauda epididymes, indicating ongoing sperm production. “Late reproduction” bats were generally very aggressive during feeding and handling (personal observation), potentially because of elevated testosterone levels (Racey 1974), which may be involved in lower torpor use by male bats during reproduction (Speakman et al. 2003). The pattern of decreased torpor use in “late reproduction” bats fits observations in free-ranging temperate zone male Daubenton’s bats (*Myotis daubentonii*, Becker et al. 2013) and captive male little brown bats (*Myotis lucifugus*) that experienced natural roosting conditions (Kurta and Kunz 1988).

Torpor is often associated with low *T*_a_ (Geiser 2004). However, there is increasing evidence that torpor can be used at higher *T*_a_ in different bat species (O'Mara et al. 2017a; Reher and Dausmann 2021), including female *N. noctula* (Keicher et al. 2022). Our male *N. noctula* decoupled *f*_H_ and V̇O_2_ from *T*_lab_ to *T*_skin_ during torpor. While *T*_skin_ changed with *T*_lab_, no corresponding change in *f*_H_ or V̇O_2_ was observed (Fig. 1A, B, and D). We observed the same pattern in free-ranging, torpid bats. They maintained a constantly low *f*_H_ at field *T*_a_ between 10 and 20°C. Thus, it appears that both sexes of *N. noctula* are capable of maintaining this low rate of metabolism even when exposed to a non-natural temperature regime (from 0° to 32.5° in the “calibration” experiment; Supplementary Fig. S1; Keicher et al. 2022), and irrespective of their life history stage. As this is a recent finding, the maximum ambient temperature at which *N. noctula* can remain torpid, as well as if this phenomenon is a universal feature of temperate-zone bats both remain unknown. Further, the underlying physiological mechanisms controlling transitions to and from torpor, and the environmental and social circumstances under which high-temperature torpor is used remain largely unexplored.

We wanted to know if the torpor use behavior in the laboratory is representative of the behavior in the field. Captive and free-ranging “pre-reproduction” bats used the same energy-saving strategy and used torpor >75% of the day (Fig. 3A) and ∼50% of the night (Fig. 3B). At this stage, mean *T*_lab_ were lower and more stable than mean *T*_a_ in the field. Even though *T*_a_ is thought to be an important factor influencing torpor use in some bat species (Wojciechowski et al. 2007; Fjelldal et al. 2021), it did not seem to affect the relative time “pre-reproduction” bats spent torpid. In contrast, “early reproduction” bats experienced the same mean *T*_a_ in captivity and in the wild but did not use the same energy-saving strategies. In the field, bats avoided daytime torpor, potentially to promote spermatogenesis, even though the same individuals had behaved differently in the laboratory, where all except two of the bats used torpor for >75% of the day. It should be noted that *T*_lab_ were generally less variable and more stable compared to *T*_a_ in the field. This could potentially affect torpor use behavior (Audet and Fenton 1988). However, as we were not able to obtain *T*_a_ directly from roosts, in which *T*_a_ are often more stable compared to the outside, but can also increase drastically, for example, if the bat box faces the sun, we can only speculate about fluctuations in *T*_a_ in the roost and their possible effects on torpor use. Generally, our data suggests that “early reproduction” bats still flexibly used torpor, most likely because conditions were not optimal for them in the laboratory (Komar et al. 2022).

The social conditions and feeding behavior differed between the laboratory and the field during “early reproduction” but not “pre-reproduction”, potentially causing the observed differences in daytime torpor use behavior. Free-ranging “pre-reproduction” male *N. noctula* roosted mainly solitarily and foraged only once per night, conditions that were matched in the laboratory. In contrast, free-ranging “early reproduction” bats in our study area formed short-lived colonies of 25–30 individuals in summer and likely used social thermoregulation during spermatogenesis to save energy (Dietz and Kalko 2006; Safi 2008). “Early reproduction” bats foraged several times and almost twice as long as “pre-reproduction” bats each night, presumably to increase food uptake and balance higher energy expenditure (Dietz and Kalko 2007; Becker et al. 2013). In contrast, we fed our captive bats only once in the evening and housed them individually in respirometry chambers. Food consumption in the laboratory did not change between life history stages (Table 1). However, this only reflected how much the bats would have consumed during their first foraging flight, and captive bats may have stopped eating once their stomach capacity was reached but would have fed again later if permitted. Indeed, captive “early reproduction” bats aroused more frequently and rested during the night, potentially reflecting their natural foraging pattern, and the time spent torpid did not differ from the free-ranging bats during the night (Figs. 3B, Supplementary Fig. S2B and D). After each foraging flight and feeding in the laboratory, bats did not enter torpor for at least 1 h, likely to digest the consumed insect prey (Matheson et al. 2010). We suggest that, because of the higher thermoregulation effort when roosting alone and because they could not increase food uptake, “early reproduction” bats decided to enter torpor in the laboratory, even though this came at the cost of slowed down sperm production (Kurta and Kunz 1988).

When interpreting differing results from the laboratory and field, it is important to distinguish between long- and short-term studies. For example, studies comparing torpor or hibernation across several months show that torpor use is most often decreased or even avoided under laboratory conditions (McNab 1982; Geiser et al. 2000). These differences can be even more pronounced in captive-bred animals or wild-caught individuals that have been in captivity for a long time and which might show changes in body mass or activity (Geiser and Ferguson 2001; Geiser et al. 2007). Thus, torpor use/hibernation duration may be highly underestimated when laboratory data are used to predict behavior in the wild over a longer time period (Geiser et al. 2000). In our study, bats were caught a maximum of one day before the onset of the laboratory experiment, and effects of long-term captivity or differences in body mass can be excluded. However, we cannot exclude that stress induced by short-term captivity, handling, or transmitter attachment (O'Mara et al. 2014) affected torpor use, and it is likely that stress responses differ across life history stages and/or individuals. Similar to our bats, male long-eared bats (*Nyctophilus geoffroyi*) often remained more active in the field while they were torpid at a more constant *T*_lab_ (Geiser et al. 2000). However, in that study, *T*_a_ influenced torpor use, and *T*_lab_ were not adjusted to match those in the field, which makes the lab/field comparison more challenging. The main conclusion based on accumulating evidence is, however, that heterothermic bat species can use torpor flexibly and adjust their metabolism to current social and/or environmental conditions at each reproductive stage. In combination with individual stress responses to captivity, this likely explains most of the discrepancies between lab/field observations in short-term torpor studies.

Our study adds to the growing literature about differing findings in laboratory and field studies investigating torpor (Geiser et al. 2000) and other behavioral and physiological parameters (Costa and Sinervo 2004; Calisi and Bentley 2009). This highlights that extrapolations from the laboratory to the field have to be made carefully. However, the combination of laboratory and field observations helped us to identify possible factors that affect torpor use strategies of *N. noctula* in the context of reproduction. Furthermore, it made it possible to detect the bats’ capability of decoupling V̇O_2_ and *f*_H_ from *T*_skin_ over a wide range of *T*_lab_ in the laboratory setting. The use of heart rate transmitters and other technological advances in biologging devices may open many opportunities for the detailed study of animal behavior in the wild (Williams et al. 2021; Jetz et al. 2022; Wild et al. 2022), but at least in the near future, physiological parameters still need to be calibrated at high resolution in the laboratory. Combined laboratory and field approaches will therefore be a powerful tool to fully understand how animals use survival and energy management strategies and how they adjust their physiology and behavior to environmental conditions in their natural habitats across life history stages.

## Supplementary Material

icad040_Supplemental_File

## Data Availability

The data underlying this article are available in the Open Research Data Repository of the Max Planck Society “Edmond” (https://doi.org/10.17617/3.NRNSSH).

## Notes

From the symposium “Daily torpor across birds and mammals: Recent progress and how do we advance the field?” presented at the annual meeting of the Society for Integrative and Comparative Biology in Austin, Texas from January 3-7, 2023.
