# Supplementary material for: Contrasting Torpor Use by Reproductive Male Common Noctule Bats in the Laboratory and in the Field
Source: Integr Comp Biol. 2023 May 26;63(5):1087–98. doi: 10.1093/icb/icad040 (PMC10714913; doi:10.1093/icb/icad040)
Supplement: icad040_Supplemental_File [file icad040_supplemental_file.docx]

Supplemental Information

**Detailed description of procedures and methods**

***Heart rate transmitter attachment and monitoring***

We attached external heart rate transmitters (ca. 0.8 g, 5 x 3 x 8mm; SP2000 HR Sparrow Systems, Fisher, Illinois, USA) at least six hours before the first experiment (Keicher et al. 2022; O'Mara et al. 2014). Each transmitter emits a continuous signal interrupted by cardiac muscle potentials. Transmitters were sewed to fabric with two lead wires extending through. We cut fur between the shoulder blades and in the left lumbar region at the two wire insertion points. We then punctured the disinfected skin, inserted the wire leads ca. 5mm, glued them in place with surgical cement (Perma-Type Company, Plainville, Connecticut, USA) and then glued the fabric with the mounted transmitter to the back, covering the wire insertion points. The glue detached after a maximum of seven days and the wires were pulled out when the transmitter fell off without observable negative consequences for the bats. We recorded f_H_ continuously with receivers (AR8000, AOR Ltd, Tokyo, Japan) connected to digital recorders (Tascam DR-05, Los Angeles, California, USA). In the field we placed receivers and recorders in a plastic box in close proximity to the bat roost and attached both devices to a portable power generator (Beaudens, Kaluojie E-commerce Co. Ltd, Shenzhen, China) to ensure continuous recording.

We used a custom R script to automatically identify the interruptions of the carrier signal by the muscle potentials and calculate f_H_ in beats per minute (bpm; Keicher et al. 2022; O'Mara et al. 2017a; O'Mara et al. 2017b). Automatically analyzed files were visually subsampled frequently to validate the filtering method, particularly when variation in f_H_ was high. One observer (LK) manually counted heartbeats when automated analysis was not possible due to interference or noise.

***Skin temperature monitoring***

In the laboratory we measured T_skin_ with iButtons (ca. 1.6 g, DS1922L, Maxim Integrated Products, San Jose, California, USA) modified following Lovegrove (2009). The iButtons recorded T_skin_ every two minutes. We glued them with surgical cement near the place of insertion of the lower wire where fur was already removed. After completing the laboratory experiments, we removed iButtons immediately.

***Respirometry setup and calculation of oxygen consumption***

To measure metabolic rates, we used an open-flow pull through respirometry system with humidity control (Sable Systems International, Las Vegas, NV, USA). This setup allows for simultaneous analysis of O_2_, CO_2_ and water vapor pressure (WVP) from up to three individuals plus one empty control chamber for collecting baseline values. We zeroed and spanned the setup before each sampling season using laboratory reference gases. Bats were placed in small airtight plastic containers (volume = 800 mL) equipped with an iButton (DS1922L, Maxim Integrated Products, San Jose, California, USA) to monitor temperatures directly in the container, and a plastic grid wrapped in mesh. The grid allowed the bats to roost in a natural hanging position while allowing air circulation. Four mass flow systems (MFS) with mass flow meters pulled humidity-controlled air (DG-4) through a copper spiral for faster temperature equilibration with a constant flow rate of 150 mL/min through each of the four chambers. A subsampler (RM-8) switched between chambers every three minutes and O_2_, CO_2_ and WVP were analyzed with a field metabolic system (FMS). We placed the respirometry chambers and the copper spiral into a climate-controlled incubator (KB53, Binder GmbH, Tuttlingen, Germany) with a small opening on the lid to mimic the roost or bat box entrance and set the light regime in the room to the natural local circadian rhythm.

Individual rates of O_2_ consumption and CO_2_ production were calculated using equations 11.7 and 11.8 from Lighton et al. (2018). The first 30 seconds were removed after a channel switch, and we corrected for drift using a spline fit (Forsythe 1977), and phase-matched the raw O_2_ and CO_2_ data. We corrected for water vapor dilution, calculated in- and excurrent fractional gas concentrations and dry corrected using equation 8.6 from Lighton et al. (2018). We standardized V̇O_2_ with the mean of the body mass before and after experiments and report V̇O_2_ in mL O_2_ g^-1^ h^-1^.

| **Table S1:** Overview of sample sizes in the different laboratory experiments and in the field heart rate monitoring across life history stages and different years. Note that the sample sizes include all tagged individuals, but that final sample size in the field was lower due to missing data from some individuals. Final sample sizes are given in parentheses. | | | | | |  |
| --- | --- | --- | --- | --- | --- | --- |
| Life history stage (year) | Laboratory | | | Field | |  |
|  | Day  (Heart rate and VO_2_) | Night  (Heart rate) | Calibration (Heart rate and VO_2_) | Day  (Heart rate) | Night  (Heart rate) | |
| Pre-reproduction (2019) | 4 | - | 3 | - | - |  |
| Pre-reproduction (2020) | 3 | 7 | 7 | 7 (4) | 7 (3) |  |
| Early reproduction (2020) | 9 | 9 | 9 | 9 (4) | 9 (5) |  |
| Late reproduction (2019) | 7 | 7 | 7 | - | - |  |
| Post reproduction (2019) | 8 | 8 | 8 | - | - |  |

**Table S2:** Relative time spent in torpor during the day and night in the laboratory across four life history stages (Beta regression output).

|  | **Relative time in torpor (day)** | | | **Relative time in torpor (night)** | | |
| --- | --- | --- | --- | --- | --- | --- |
| ***Predictors*** | ***Estimates*** | ***CI*** | ***p*** | ***Estimates*** | ***CI*** | ***p*** |
| Intercept | 3.54 | 1.53 – 8.19 | 0.003 | 1.08 | 0.60 – 1.94 | 0.809 |
| Early reproduction | 0.39 | 0.13 – 1.15 | 0.088 | 0.20 | 0.08 – 0.47 | <0.001 |
| Late reproduction | 0.07 | 0.02 – 0.24 | <0.001 | 0.11 | 0.04 – 0.28 | <0.001 |
| Post reproduction | 1.06 | 0.35 – 3.18 | 0.915 | 1.85 | 0.81 – 4.21 | 0.145 |
| Observations | 31 | | | 31 | | |
| R^2^ | 0.523 | | | 0.563 | | |

V̇O_2_ (mL O_2_ g^-1^ h^-1^)

f_H_

V̇O_2_

T_lab_

T_skin_

**A**

**B**

**Figure S1: Exemplary plot of one bat (post reproduction) in the “calibration” experiment.** **A)** f_H_ (grey solid line) and V̇O_2_ (black dashed line) remained constantly low after a short arousal at the beginning of the experiment. **B)** T_skin_ (grey solid line) increases with increasing T_lab_ (black dotted line) from 0°C to 32.5°C.

**A**

**C**

**B**

**D**

Pre-reproduction laboratory

Pre-reproduction field

Early reproduction laboratory

Early reproduction field

**Figure S2: Exemplary plot of daytime and nighttime heart rate in a “pre-reproduction” bat in the laboratory (A), an “early reproduction” bat in the laboratory (B), a “pre-reproduction” bat in the field (C), and an “early reproduction” bat in the field (D). A)** After a short arousal (orange) a captive “pre-reproductive” bat enters torpor (blue) until the end of the “laboratory day” experiment. After feeding the bat rests (pink) until it enters torpor around midnight and remains torpid until the morning. **B)** After a short arousal a captive “early reproductive” bat enters torpor and arouses just before the end of the “laboratory day” experiment. After feeding the bat rests until the morning. **C)** A free-ranging “pre-reproductive” bat is torpid throughout the day and arouses in the evening for a foraging flight. After feeding the bat rests until it enters torpor in the morning before sunrise. **D)** A free-ranging “early reproductive” bat does not enter torpor but rests throughout day and night and forages around sunset.

**References**

Forsythe GE, Malcolm M. A., and Moler C. B. 1977. Computer methods for mathematical computations.

Keicher L, Shipley JR, Komar E, Ruczyński I, Schaeffer PJ, Dechmann DKN. 2022. Flexible energy-saving strategies in female temperate-zone bats. J Comp Physiol B.

Lighton JR. 2018. Measuring metabolic rates: A manual for scientists. Oxford: Oxford University Press.

Lovegrove BG. 2009. Modification and miniaturization of thermochron ibuttons for surgical implantation into small animals. J Comp Physiol B. 179(4):451-458.

O'Mara MT, Rikker S, Wikelski M, Ter Maat A, Pollock HS, Dechmann DKN. 2017a. Heart rate reveals torpor at high body temperatures in lowland tropical free-tailed bats. R Soc Open Sci. 4(12):171359.

O'Mara MT, Wikelski M, Dechmann DKN. 2014. 50 years of bat tracking: Device attachment and future directions. Methods Ecol Evol. 5(4):311-319.

O'Mara MT, Wikelski M, Voigt CC, Ter Maat A, Pollock HS, Burness G, Desantis LM, Dechmann DKN. 2017b. Cyclic bouts of extreme bradycardia counteract the high metabolism of frugivorous bats. eLife. 6:e26686.
